# BTF3 affects hepatocellular carcinoma progression by transcriptionally upregulating PDCD2L and inactivating p53 signaling

**DOI:** 10.1186/s10020-024-01044-x

**Published:** 2024-12-20

**Authors:** Minyu Kong, Xiaoyi Shi, Jie Gao, Wenzhi Guo

**Affiliations:** 1 Department of Hepatobiliary and Pancreatic Surgery, The First Affiliated Hospital of Zhengzhou University, Zhengzhou, 450052 China; 2 Key Laboratory of Hepatobiliary and Pancreatic Surgery and Digestive Organ Transplantation of Henan Province, The First Affiliated Hospital of Zhengzhou University, Zhengzhou, 450052 China; 3 Open and Key Laboratory of Hepatobiliary & Pancreatic Surgery and Digestive Organ Transplantation at Henan Universities, Zhengzhou, 450052 China; 4 Henan Key Laboratory of Digestive Organ Transplantation, Zhengzhou, 450052 China

**Keywords:** BTF3, PDCD2L, p53, Hepatocellular carcinoma

## Abstract

Hepatocellular carcinoma (HCC) is one of the most common malignant tumors, with the characteristics of high mortality and low 5-year survival rate. The potential role of BTF3 and PDCD2L in HCC remains unclear. Our study found that BTF3 expression was upregulated in hepatocellular carcinoma tissues, and its high expression was associated with poor prognosis. Knockdown of BTF3 significantly inhibited proliferation and promoted apoptosis of hepatocellular carcinoma cells by cell function assay. Mechanistically, BTF3 plays an oncogenic role by regulating the transcriptional expression of PDCD2L, which promotes proliferation and inhibits apoptosis of HCC cells by restraining the p53 pathway. In conclusion, our results suggest that BTF3 induces malignant progression of HCC by acting as a transcription factor that promotes the transcription of PDCD2L and influences the p53 pathway and that the BTF3/PDCD2L/P53 axis may be a future therapeutic strategy for HCC patients.

**Supplementary Information:**

The online version contains supplementary material available at 10.1186/s10020-024-01044-x.

## Introduction

Transcription factors regulate many oncogenes in cancer, increasing their expression (Cao et al. 2024; Yao et al. 2024). Therefore, it is interesting to investigate how transcription factors aberrantly expressed in liver cancer regulate oncogenes to induce liver cancer development. Basic transcription factor 3 (BTF3) is a 27 kDa protein initially reported to form a transcriptionally active complex with RNA polymerase II (Zheng et al. 1987). It has been found that the expression of BTF3 is increased in a variety of cancers, including prostate, gastric (Zhang et al. 2017), breast (Ding et al. 2019; Wang et al. 2021), pancreatic (Kusumawidjaja et al. 2007), colorectal (Wang et al. 2020), and liver cancers (Roy et al. 2010). BTF3 mainly acts as a transcription factor to regulate the expression of oncogenes, but it has been shown that BTF3 can also restrict the translation of proteins (Hu et al. 2019). BTF3 has been shown to regulate the proliferation, migration, and invasion of prostate cancer and DNA damage repair (Liu et al. 2019; Wu et al. 2020; Zhang et al. 2021). Recent studies have shown that BTF3 can promote glycolysis in hepatocellular carcinoma cells (Wang et al. 2023). However, given the transcriptional activity of BTF3, the mechanism by which BTF3 regulates hepatocellular carcinoma development remains unclear.

Programmed Cell Death, 2 Like (PDCD2L) is an encoded protein initially shown to induce apoptosis in pancreatic β-cells (Yin et al. 2016). Still, there have been few studies of PDCD2L in cancer, with only one study confirming that PDCD2L expression increases and promotes the proliferation of colorectal cancer cells. That knockdown of PDCD2L promotes apoptosis in colorectal cancer cells (Gao et al. 2022).

In the current study, we investigated the biological role of BTF3 in hepatocellular carcinoma and confirmed that PDCD2L is a downstream target of BTF3 as a transcription factor. We also investigated the natural role of PDCD2L in hepatocellular carcinoma and its mechanism. Finally, our study demonstrated the possibility of treating hepatocellular carcinoma by targeting the BTF3/PDCD2L/p53 axis.

## Results

### BTF3 is upregulated in HCC tissue and associated with the prognosis of patients

**Fig. 1:**
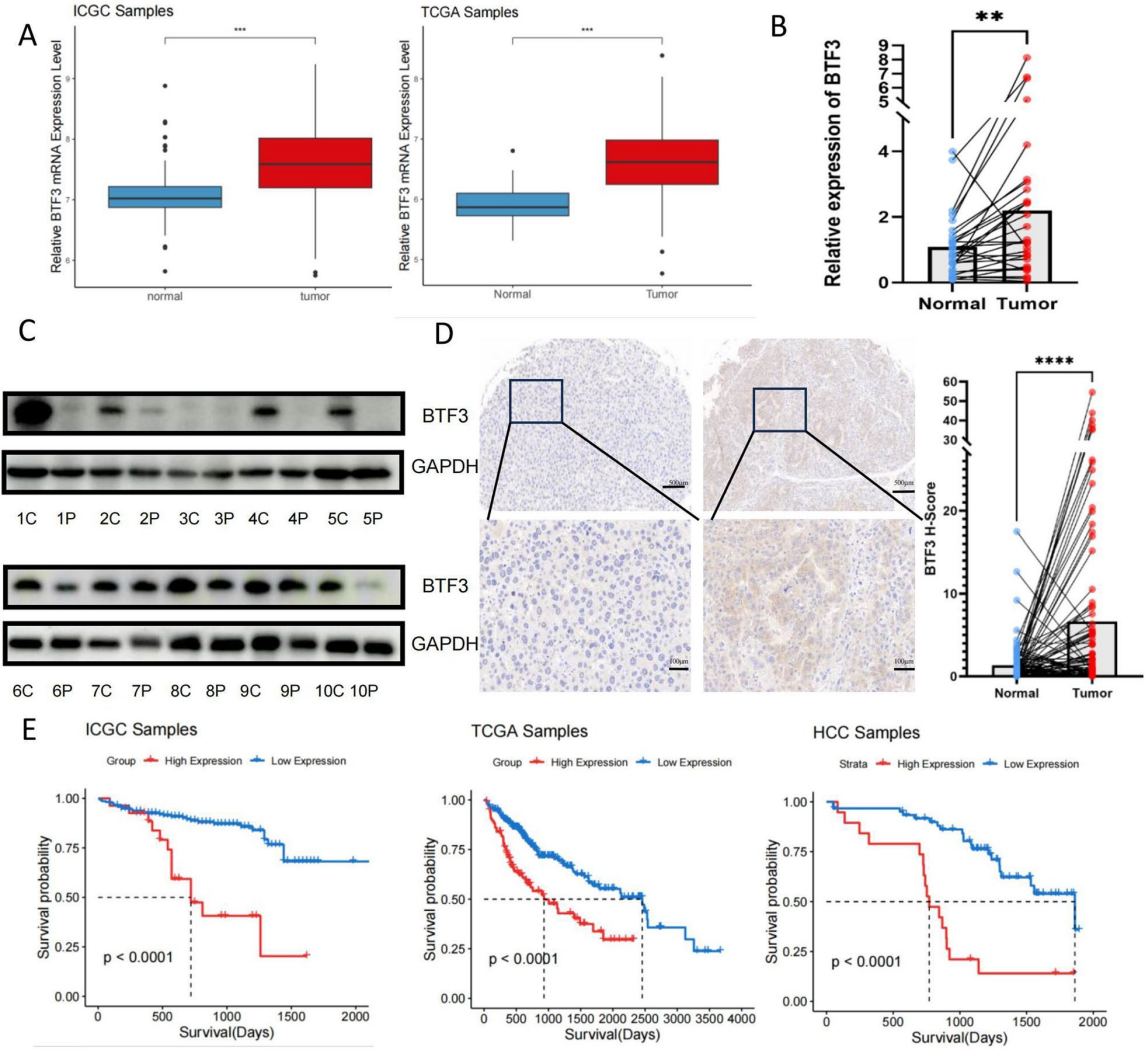
BTF3 is highly expressed in hepatocellular carcinoma tissues and correlates with poor patient prognosis. **A** The mRNA expression of BTF3 in hepatocellular carcinoma tissues was significantly higher than that in normal tissues as derived from the ICGC dataset, ***p < 0.001 ((Student's t-test)). **B** BTF3 expression in 29 pairs of hepatocellular carcinoma tissue samples was detected by qRT-PCR, **p < 0.01 ((Student's t-test). **C** BTF3 protein expression was detected by Western blot in 10 pairs of liver cancer tissues. **D** BTF3 expression was detected by immunohistochemistry in 89 pairs of hepatocellular carcinoma tissues, and BTF3 was found to be significantly highly expressed in cancer tissues by H-Score ****p < 0.0001 (Student's t-test). **E** Using HCC patients from ICGC, TCGA, and immunohistochemistry samples, the survival R package determined the optimal cut-off value of BTF3 expression and divided into low and high-expression groups. Subsequently, the overall survival rates of the two groups were compared by Kaplan–Meier survival analysis (p < 0.0001)

To determine the expression level of BTF3 in HCC tissues, we analyzed the mRNA expression level of BTF3 and the prognosis of BTF3 using HCC data from the TCGA and ICGC databases (Fig. 1A, E). The results of the TCGA and ICGC data indicated that BTF3 was significantly up-regulated in HCC tissues and that high expression of BTF3 was correlated with a poor prognosis of patients. Subsequently, we extracted mRNA from 29 pairs of hepatocellular carcinoma tissues for qRT-PCR, and the results showed that the expression of BTF3 in hepatocellular carcinoma tissues was significantly higher than that in paired paracellular carcinoma tissues (Fig. 1B). Meanwhile, we extracted proteins from 10 pairs of hepatocellular carcinoma tissues for western blot experiments, among which seven pairs of tissues showed significantly high expression of BTF3 in cancerous tissues (Fig. 1C). In addition, we also analyzed BTF3 expression in 89 pairs of HCC tissues using immunohistochemistry, and the results were consistent with the trend of PCR in 29 pairs of hepatocellular carcinoma tissues (Fig. 1D). Survival analysis using the Kaplan–Meier method showed that higher BTF3 expression predicted shorter OS time in 89 patients with HCC (Fig. 1E). In conclusion, these data suggest that BTF3 is highly expressed in hepatocellular carcinoma tissues and is significantly correlated with a worse prognosis in patients with HCC.

### In vitro BTF3 promotes proliferation and inhibits apoptosis in hepatocellular carcinoma cells

**Fig. 2:**
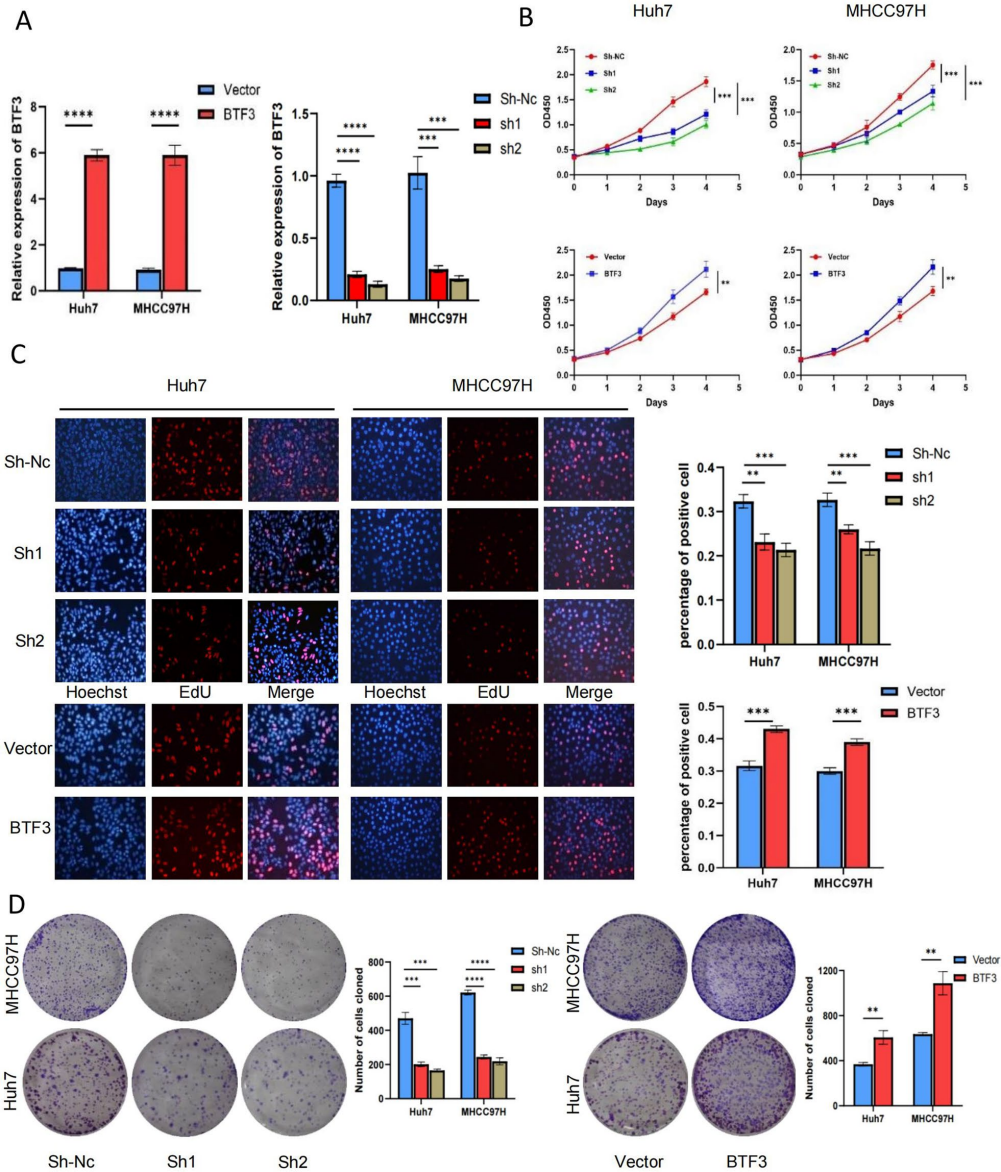
BTF3 promotes HCC cell proliferation in vitro. **A** Huh7 and MHCC97H cells were transfected with BTF3 knockdown or overexpression plasmids, and qRT-PCR verified the knockdown and overexpression efficiency. **B**–**D** BTF3 knockdown, overexpression, and control plasmids were transfected into hepatocellular carcinoma cells, and the proliferative activities of the cells were detected by CCK-8, Edu, and clone formation assays (**p < 0.01, ***p < 0.001, ****p < 0.0001 by Student's t-test)

**Fig. 3:**
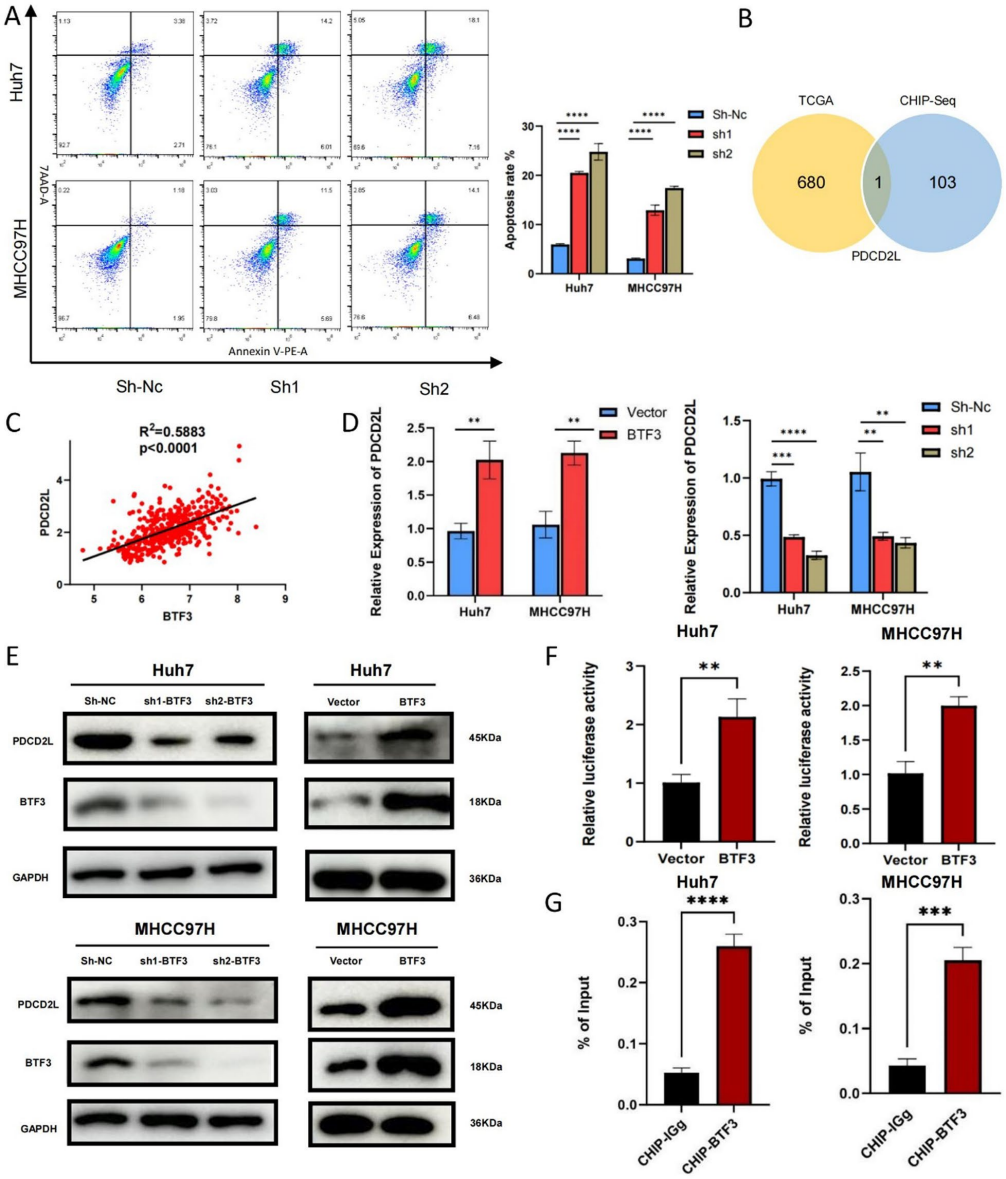
BTF3 inhibits apoptosis in HCC cells in vitro, and BTF3 directly promotes PDCD2L transcription. **A** Flow cytometry was performed to detect the effect of BTF3 down-regulation on apoptosis in Huh7 and MHCC97H cells (****p < 0.0001 by Student's t-test). **B** Venn plots based on TCGA and CHIP-seq data show one candidate target of BTF3, PDCD2L. **C** Correlation analysis of BTF3 and PDCD2L based on TCGA dataset. **D**, **E** QRT-PCR and Western blot detected changes in the expression of PDCD2L in BTF3 knockdown and overexpression cells. **F** PDCD2L promoter luciferase reporter assays were performed in Vetcor or BTF3 transfected Huh7 and MHCC97H cells (**p < 0.01 by Student's t-test). **G** Chromatin immunoprecipitation (ChIP) assay was performed using BTF3 chromatin immunoprecipitation assay and qPCR on Huh7 and MHCC97H cells. Enrichment of the PDCD2L promoter region was normalized to input. IgG was used as a negative control (***p < 0.001, ****p < 0.0001 by Student's t-test)

To investigate the potential function of BTF3 in hepatocellular carcinoma cells, we transfected MHCC97H and Huh7 cells with knockdown/overexpression plasmids of BTF3 and verified the changes of BTF3 expression in both cells by qRT-PCR. (Fig. 2A). We found that knockdown of BTF3 significantly inhibited the proliferation of hepatocellular carcinoma cells, and overexpression of BTF3 promoted the proliferation of hepatocellular carcinoma cells by CCK8 assay (Fig. 2B). First, EDU assay showed that the proportion of proliferative cells in BTF3 knockdown hepatocellular carcinoma cells was significantly reduced (Fig. 2C). In addition, colony formation assay also confirmed that BTF3-knockdown MHCC97H and Huh7 cells formed substantially fewer colonies (Fig. 2D). The above three experiments concluded that knockdown of BTF3 significantly inhibited the proliferation of hepatocellular carcinoma cells. At the same time, overexpression of BTF3 promoted the proliferation of hepatocellular carcinoma cells. In addition, flow cytometric analysis showed that the apoptosis rate of hepatocellular carcinoma cells with BTF3 knockdown was significantly increased (Fig. 3A). All these data indicate the oncogenic role of BTF3 in hepatocellular carcinoma cells.

### BTF3 directly promotes PDCD2L transcription

To investigate the specific mechanism of BTF3 acting as a transcription factor in regulating liver cancer, 680 genes positively related to BTF3 with a correlation coefficient > 0.5 were obtained from the transcriptome data of 424 cases of hepatocellular carcinoma in TCGA, and 103 genes related to BTF3 were obtained from the data of ChIP-Seq (Chromatin Immunoprecipitation Sequencing) of BTF3 in colorectal cancer. The intersection of these data was obtained as one gene (Fig. 3B). PDCD2L. The TCGA data showed that the correlation coefficient between BTF3 and PDCD2L was 0.5883 (Fig. 3C). To confirm the relationship between BTF3 and PDCD2L, we first observed the expression level of PDCD2L in hepatocellular carcinoma cells knocking down and overexpressing BTF3, and the results showed that the expression of PDCD2L was in the same trend of change as that of BTF3 (Fig. 3D, E). To further confirm that BTF3 acts in the promoter region of PDCD2L, we constructed a luciferase reporter gene plasmid based on DNA sequences between -2000 and 0, the PDCD2L transcription start site. Dual luciferase reporter gene assay showed that overexpression of BTF3 significantly enhanced pGL3-PDCD2L activity in Huh7 and MHCC97H cells, suggesting that BTF3 can act on the PDCD2L promoter and promote PDCD2L transcription (Fig. 3F). Subsequently, we verified the binding ability of BTF3 to PDCD2L promoter DNA by CHIP assay. We found that compared with IgG, the BTF3 antibody could effectively bind to the DNA fragment of the PDCD2L proximal promoter (Fig. 3G). In conclusion, the above results demonstrated that BTF3 could bind to the promoter region of PDCD2L and promote the up-regulation of PDCD2L transcription in hepatocellular carcinoma cell lines.

### In vitro PDCD2L promotes hepatocellular carcinoma cell proliferation and inhibits apoptosis

**Fig. 4:**
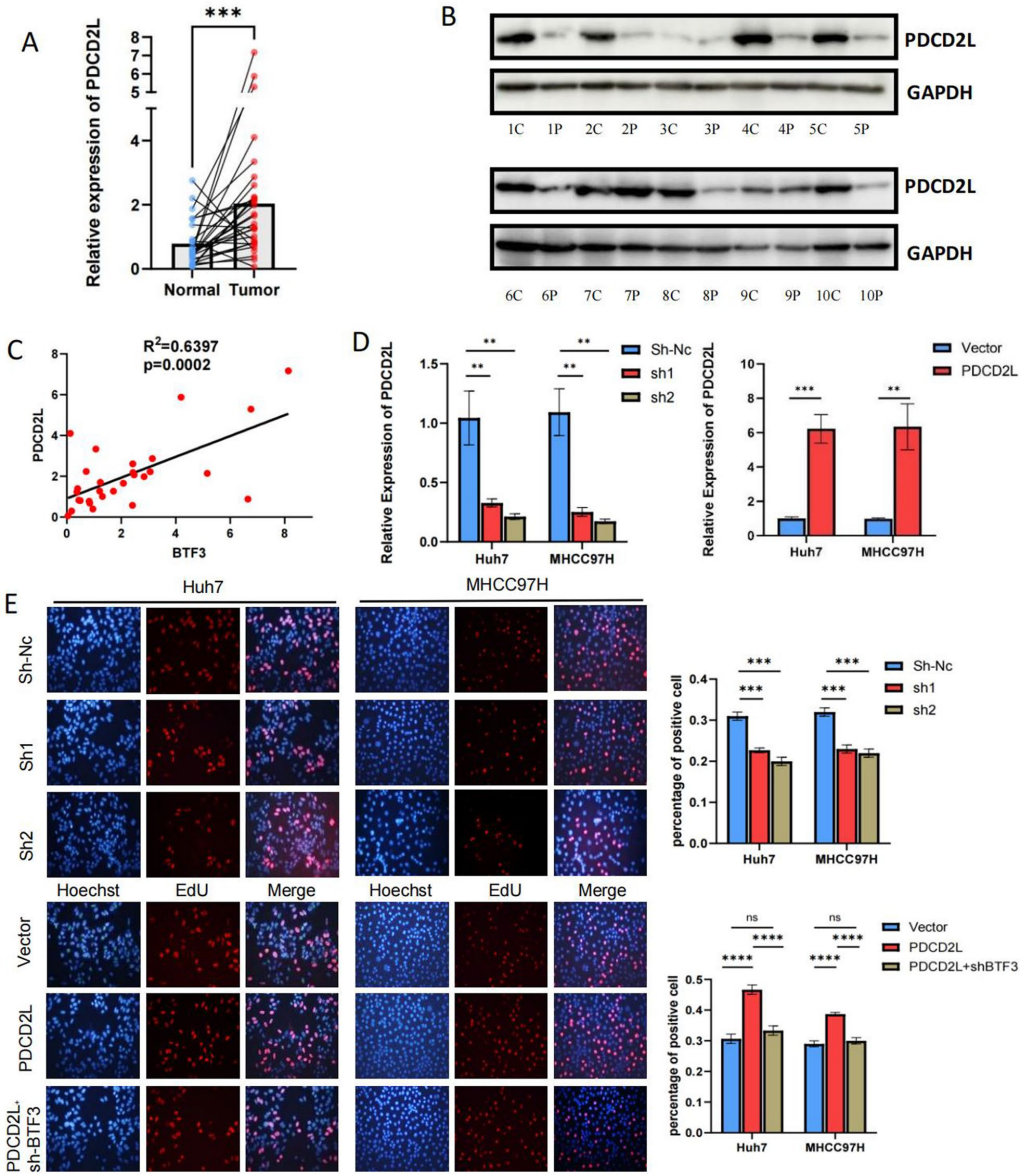
PDCD2L is highly expressed in hepatocellular carcinoma tissues and promotes cell proliferation in HCC. **A**, **B** Detection of PDCD2L expression in hepatocellular carcinoma tissues by qRT-PCR and Western blot. **C** Correlation between PDCD2L and BTF3 expression in 29 hepatocellular carcinoma tissues. **D** Transfection of BTF3 knockdown or overexpression plasmid into Huh7 and MHCC97H cells were transfected with BTF3 knockdown or overexpression plasmids, and qRT-PCR verified the knockdown and overexpression efficiency. **E** The proliferative activities of HCC cells were compared by Edu assay with up-regulation, down-regulation of PDCD2L, and up-regulation of PDCD2L combined with down-regulation of BTF3 (***p < 0.001 by Student's t-test and one-way ANOVAs)

**Fig. 5:**
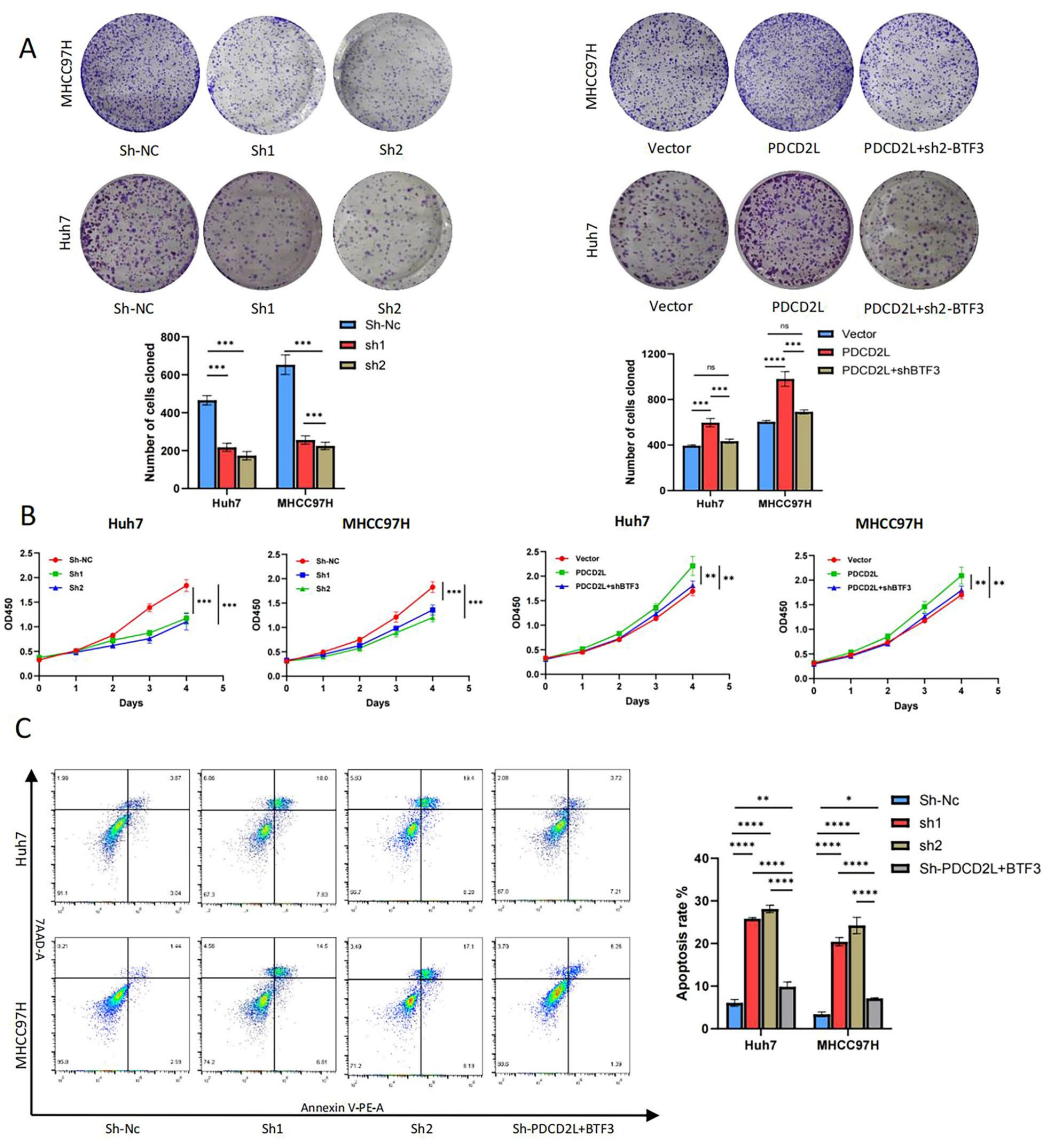
In vitro BTF3 promotes hepatocellular carcinoma cell proliferation and inhibits apoptosis via PDCD2L. **A**, **B** The proliferative activities of HCC cells upon up-regulation, down-regulation of PDCD2L, and combination of up-regulation and down-regulation of BTF3 by BTF3 were examined by clone formation and CCK-8 assays (**p < 0.01, ***p < 0.001 by Student's t-test and one-way ANOVAs). **C** Flow cytometry detection of apoptosis rate of HCC cells upon down-regulation of PDCD2L, and down-regulation of PDCD2L combined with up-regulation of BTF3 (**p < 0.01, **p < 0.01,****p < 0.0001 by one-way ANOVAs)

To investigate the role of PDCD2L in hepatocellular carcinoma, we first verified that the mRNA and protein expression of PDCD2L in hepatocellular carcinoma tissues was significantly higher than that in paracellular carcinoma tissues (Fig. 4A, B). Meanwhile, correlation analysis revealed that the expression of PDCD2L was highly correlated with that of BTF3 in 29 pairs of hepatocellular carcinoma tissues, which indirectly indicated the regulatory relationship between BTF3 and PDCD2L (Fig. 4C). Subsequently, we knocked down the overexpression of PDCD2L in hepatocellular carcinoma cell lines. We verified the changes of PDCD2L expression in hepatocellular carcinoma cell lines after transfection by q-rtPCR (Fig. 4D). To ascertain the effect of PDCD2L on the proliferation of hepatocellular carcinoma cells, we performed CCK8, EDU, and clone formation assays, which showed that knockdown of PDCD2L significantly inhibited the proliferation of hepatocellular carcinoma cells. At the same time, overexpression of PDCD2L promoted the activity of hepatocellular carcinoma cells while knockdown of BTF3 could partially reverse this trend (Figs. 4E, 5A, B). The results showed that knockdown of PDCD2L significantly inhibited the proliferation of liver cancer cells. Meanwhile, knockdown of PDCD2L promoted apoptosis in hepatocellular carcinoma cells as detected by flow cytometry, and this could be rescued by BTF3 overexpression (Fig. 5C). The above experiments indicate that PDCD2L plays a positive role in regulating the progression of hepatocellular carcinoma, similar to the role of BTF3.

### Up-regulated BTF3 inhibits p53 signaling pathway by regulating PDCD2L

**Fig. 6:**
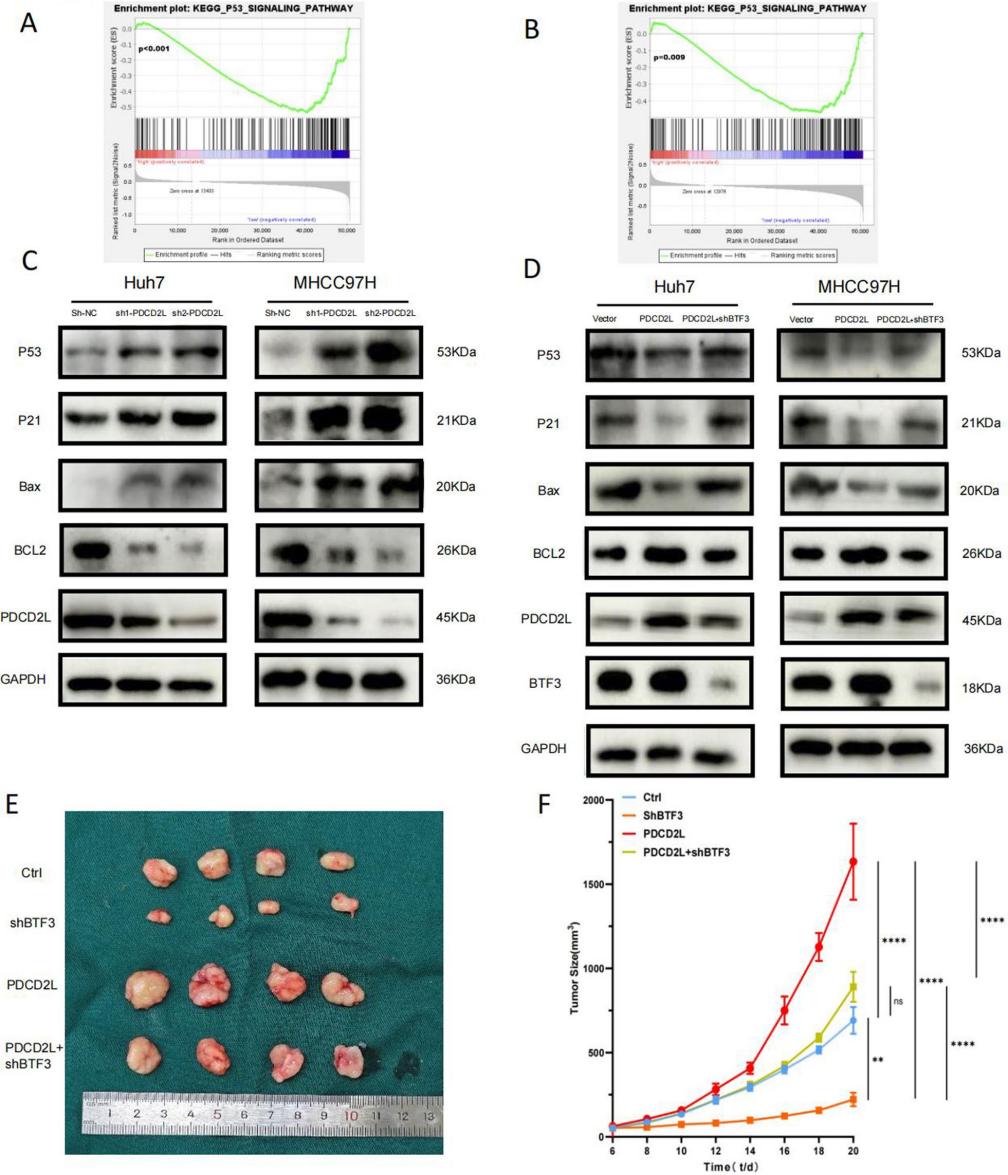
BTF3 inhibits the p53 pathway through PDCD2L. In vivo, BTF3 promotes HCC cell proliferation through PDCD2L. **A**, **B** BTF3 and PDCD2L differential genes were found to be significantly enriched in the p53 pathway by GSEA analysis. **C**, **D** Western blot assay for the expression of p53 signaling pathway-related molecules in HCC cells upon up-regulation, down-regulation of PDCD2L, and combination of up-regulation and down-regulation of PDCD2L with BTF3. **E** MHCC97H cells transfected with control, BTF3 knockdown, PDCD2L overexpression, and PDCD2L overexpression combined with BTF3 knockdown plasmid were injected into nude mice subcutaneously, and the tumor size was observed after 20 days. **F** Tumor volume was measured every two days, and tumor growth curves were plotted (***p < 0.001, ****p < 0.0001 by one-way ANOVAs)

It has been suggested that the involvement of PDCD2L in colorectal cancer progression is associated with the p53 pathway (Gao et al. 2022). We performed GSEA analysis of PDCD2L and BTF3 based on the liver cancer transcriptome data from TCGA and found that the p53 pathway was significantly enriched in the low-expression group of BTF3 and PDCD2L (Fig. 6A,B). This suggests an interaction between BTF3 and PDCD2L and possibly the p53 pathway in hepatocellular carcinoma. We first knocked down PDCD2L in two p53-mutant hepatocellular carcinoma cells (MHCC97H and Huh7) and found that the expression of p53, p21, and bax was increased, and the expression of bcl2 was decreased, while the overexpression of PDCD2L was vice versa. In addition, we knocked down BTF3 while overexpressing PDCD2L and found that the expression trend of p53-related molecules was similar to that of the control group (Fig. 6C, D). This suggests that BTF3 may regulate the p53 signaling pathway by regulating PDCD2L. Subsequently, to verify whether similar effects also occur in p53 wild-type hepatocellular carcinoma cells, we performed studies in HepG2 cells. We found that knockdown of PDCD2L in HepG2 cells revealed that knockdown of PDCD2L significantly inhibited the proliferation and promoted apoptosis of HepG2 cells (Figure S1A, B). In addition, the protein levels of p53 signaling pathway in HepG2 cells after PDCD2L knockdown showed the same trend as in MHCC97H and Huh7 cells (Figure S1C). This suggests that PDCD2L exerts its tumor-promoting function at least in part through the p53 signaling pathway in wild-type and both p53-mutant cell lines.

### BTF3 promotes hepatocellular carcinoma cell growth in vivo by regulating PDCD2L

To analyze the role of BTF3 and PDCD2L in HCC in vivo, we injected four groups of MHCC97H cells, namely, control, shBTF3, PDCD2L, and PDCD2L + shBTF3, into the subcutaneous skin of nude mice, which showed that the rhabdomyolysis of nude mice in the shBTF3 group was significantly reduced compared with that of control, and that of nude mice in the PDCD2L group was enlarged considerably. Meanwhile, the rhabdomyolysis of nude mice in the PDCD2L + shBTF3 group was significantly smaller than in the PDCD2L group (Fig. 6E, F). The above results indicated that BTF3 and PDCD2L promoted the proliferation of hepatocellular carcinoma in nude mice, and the knockdown of BTF3 could inhibit the promotion of hepatocellular carcinoma by PDCD2L.

## Discussion

Various transcription factors are essential in regulating hepatocellular carcinoma development, drug resistance, and metastasis (Kong et al. 2023; Li et al. 2010; Tarocchi et al. 2011). BTF3, as an oncogenic transcription factor, is involved in various cellular processes in prostate, colorectal, esophageal, breast, and hepatocellular carcinomas, including DNA damage repair, cell stemness, proliferation, invasion, migration, and glycolysis. Many molecules are involved in regulating BTF3 in the progression of various cancers. Among them, BTF3 promotes the proliferation of hepatocellular carcinoma and gastric cancer cells by up-regulating FOXM1 (Wang et al. 2023; Zhang et al. 2017). FXOM1, a common transcription factor regulating proliferation, has been shown to play a role in various cancers (Tang et al. 2023; Zhang et al. 2023a, b). Additionally, BTF3 acts in prostate cancer by inhibiting the degradation of BMI1 (Hu et al. 2019). BMI1 mainly maintains cell stemness to promote cell proliferation and migration during invasion in various cancer cells (Editors 2023; Yan et al. 2021). The mechanism of oncogenesis of BTF3 as a transcription factor in hepatocellular carcinoma is still unclear. The mechanism of BTF3 oncogenesis as a transcription factor in hepatocellular carcinoma is still unknown. In this study, we identified a novel role of BTF3 in hepatocellular carcinoma through transcriptional up-regulation of PDCD2L involved in the proliferation and apoptosis of hepatocellular carcinoma cells.

In the current study, we first observed the expression level and prognosis of BTF3 in hepatocellular carcinoma tissues, and the results showed that BTF3 was highly expressed in hepatocellular carcinoma tissues and correlated with the prognosis of patients. Subsequently, we obtained that BTF3 promoted the proliferation and inhibited the apoptosis of hepatocellular carcinoma cells through the overexpression of BTF3 in hepatocellular carcinoma cells. To clarify the mechanism of BTF3 as a transcription factor in hepatocellular carcinoma, we then utilized the chip-seq data and transcriptome data to identify potential targets of BTF3 and finally focused on the PDCD2L molecule.

Programmed Cell Death 2 Like (PDCD2L) is closely related to apoptosis and proliferation, but there are few studies on PDCD2L in cancer; only one study reported that PDCD2L is highly expressed in colorectal cancer and promotes cell proliferation and inhibits apoptosis in colorectal cancer. The role of PDCD2L in hepatocellular carcinoma needs to be further studied. Our work shows that BTF3 can directly act on the promoter of PDCD2L to promote the transcription of PDCD2L and exert oncogenic effects in hepatocellular carcinoma. We also demonstrated that PDCD2L is highly expressed in hepatocellular carcinoma and regulates the proliferation and apoptosis of hepatocellular carcinoma cells to promote the progression of hepatocellular carcinoma. Subsequently, GSEA enrichment analysis revealed that the p53 pathway was enriched in BTF3 and PDCD2L low-expression groups. According to statistics, 60% of cancers are associated with p53, and active p53 is important in tumor suppression (Gala et al. 2024; Hernandez Borrero and El-Deiry 2021). Meanwhile, the role of the p53 pathway in regulating cell proliferation and apoptosis has been widely confirmed (Ma et al. 2023; Wang et al. 2022). Clinical trials of p53-MDM2/MDM4 antagonists are underway in cancer patients (Duffy et al. 2022). MDM2 and MDM4 are known to act as upstream molecules of p53 by regulating p53 activity (Chen et al. 2024; Mei et al. 2023). Therefore, the discovery of upstream regulators of p53 may lead to improved insights into the p53 pathway and better targeting of the p53 pathway in cancer. Our study found that BTF3 may promote PDCD2L to inhibit the p53 signaling pathway. However, we have only verified the effects of BTF3 and PDCD2L on the expression levels of p53 pathway-related molecules, and how PDCD2L acts on the p53 pathway still needs to be clarified and requires further in-depth exploration.

**Fig. 7:**
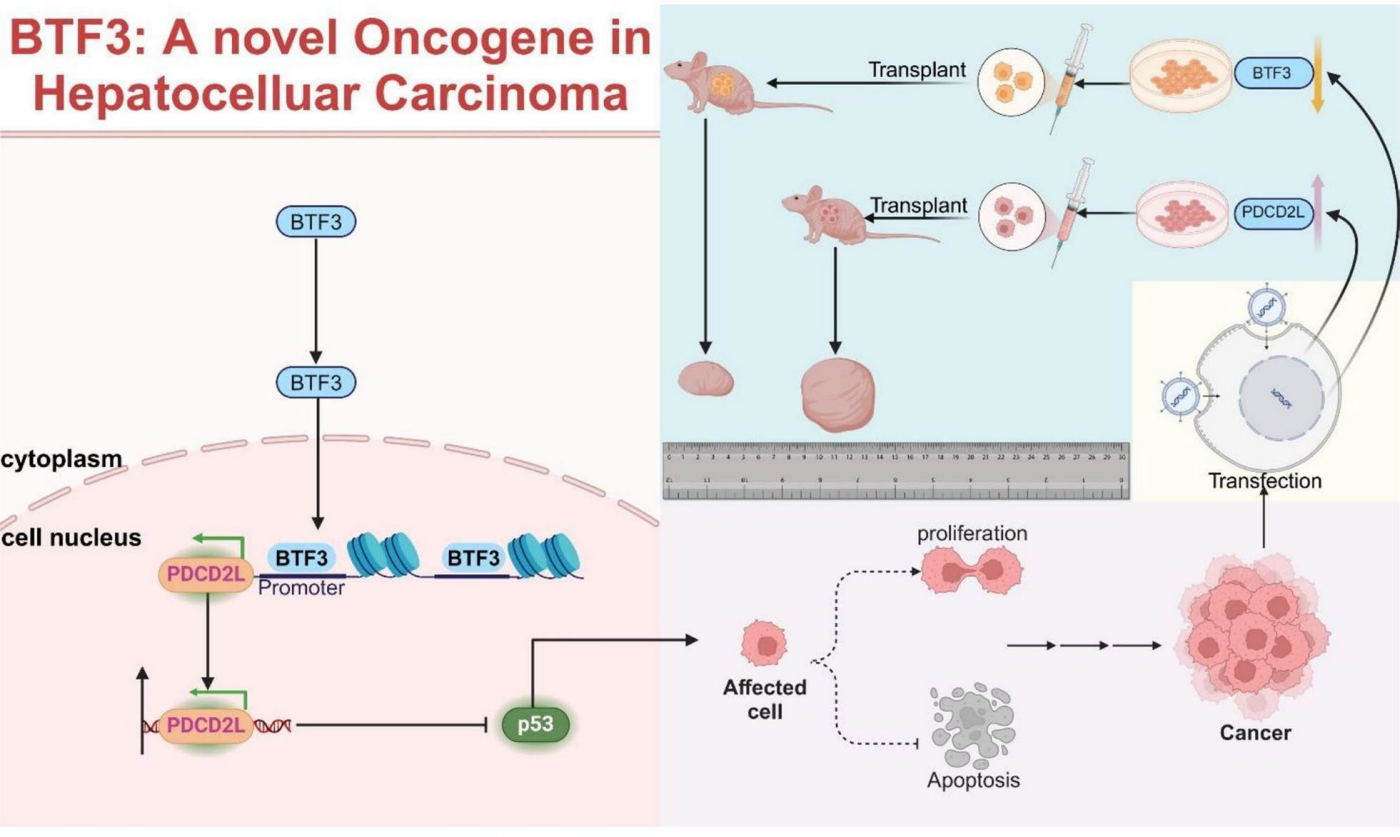
The schematic diagram of BT3-PDCD2L-p53 in regulating cell proliferation, apoptosis in HCC

In conclusion, our work identified the oncogenic role of BTF3 as an oncogenic transcription factor in hepatocellular carcinoma. Also, it confirmed the oncogenic role of PDCD2L in hepatocellular carcinoma for the first time. In hepatocellular carcinoma, BTF3 further regulates the p53 pathway through pro-transcriptional up-regulation of PDCD2L (Fig. 7), which may provide some insights into the development of hepatocellular carcinoma. Meanwhile, we provide reliable evidence for the potential application of BTF3 and PDCD2L as diagnostic and prognostic biomarkers and therapeutic targets in hepatocellular carcinoma.

## Materials and methods

### Patient information, gene expression, and survival data

Tumor tissues and paired normal liver tissues were collected from 128 pairs of hepatocellular carcinoma patients between June 2018 and June 2023. All patients signed an informed consent form, and the First Affiliated Hospital Ethics Committee of Zhengzhou University, China, approved the study protocol. Transcriptomic data were collected from the TCGA-LIHC cohort from UCSC (https://xenabrowser.net/datapages/), which consisted of 374 hepatocellular carcinoma specimens and 50 standard specimens. HCC transcriptomic data were collected from the ICGC with 243 hepatocellular carcinoma and 202 common specimens (https://dcc.icgc.org/). Gene set enrichment analysis utilizing GSEA software (http://www.broadinstitute.org/gsea). Survival information of the patients was collected, the Survival R package determined the optimal cut-off value, the data were categorized into high and low-expression groups, and further Kaplan–Meier survival analysis was completed.

### Cell culture and transfection

Two human hepatocellular carcinoma cell lines (HepG2, Huh7 and MHCC97H) were obtained from the China Center for Type Culture Collection (Wuhan, China). Cells were cultured using DMEM medium (Solarbio, China) supplemented with 1% (penicillin and streptomycin) and 10% fetal bovine serum (FBS). The temperature of the incubator was 37 °C and the CO2 content was 5%. Transfection of plasmids was performed using a lipo8000 (Beyotime, Shanghai, China) transfection reagent. Plasmid vector, BTF3, and PDCD2L plasmids were obtained from Public Protein/Plasmid Library (Public Protein/Plasmid Library, China). The two genes were knocked down using sh-BTF3 and sh-PDCD2L. The plasmid sequences used are shown in Supplementary Table 1(Table.S1).

### Western blot analysis

Cells or tissues were lysed on ice using RIPA lysate containing 1% RIPA, and samples were separated by 10% SDS-PAGE or 12% SDS-PAGE and transferred to PVDF membranes. The PVDF membrane was then blocked with a blocking buffer for 330 min and incubated sequentially with primary and secondary antibodies; see Supplementary Table 1 (Table.S1) for information on the antibodies used.

### Quantitative real-time PCR (qRT-PCR)

QuantStudio™5 Real-Time PCR System (Thermo Fisher Scientific) was used for analysis by qRT-PCR. Cycling conditions: The initial denaturation temperature was 95 ˚C for 30 s. This was followed by 40 cycles of 95 ˚C for 10 s, 60 ˚C for 30 s, and finally a dissolution curve was prepared from 60 ˚C to 95 ˚C. Fold change in gene expression was calculated by the 2 -ΔΔCT method using 18S as endogenous control. The primer sequences involved are shown in Supplementary Table 1 (Table.S1).

### CCK-8 assay

According to the guidelines, the CCK-8 kit (Solarbio, China) was used for cell viability assay. The transfected Huh7 and MHCC97H cells were homogeneously inoculated in 96-well plates, and on days 0, 1, 2, 3 and 4, respectively, 10ul of CCK-8 solution was added to the wells and the absorbance was measured. An enzyme marker measured the absorbance after incubation at 37 °C for 120 min.

### 5-Ethynyl-2′-deoxyuridine (EdU) assay

According to the guidelines, cell proliferation viability was detected using the EdU-594 Cell Proliferation Assay Kit (Beyotime, Shanghai, China). Transfected HepG2, Huh7 and MHCC97H cells were inoculated into 96-well plates and cultured for 24 h. The cells were added to the EdU solution and cultured for two hours. After that, the cells were fixed, permeabilized, and stained according to the kit guidelines. Nuclei were stained with Hoechst 33342. After staining, the cells were washed with PBS and visualized by a fluorescence microscope, and images were collected.

### Colony formation assay

The transfected Huh7 and MHCC97H cells were homogeneously inoculated into 6-well plates at a density of 1000 cells per well, cultured for two weeks, fixed with 4% paraformaldehyde, and stained with 0.1% crystal violet.

### Apoptosis assay

Apoptosis was detected using Annexin V-PE and 7-AAD (Beyotime, Shanghai, China). Transfected HepG2, Huh7 and MHCC97H cells were inoculated into 6-well plates and cultured for 48 h. Adherent and suspended cells were collected and resuspended using buffer, adding five μl of Annexin V-PE and 5 μl of 7-AAD (20×) and incubating for 15 min away from light. Then, apoptosis was detected using flow cytometry. The apoptosis rate is the sum of the early apoptosis rate and the late apoptosis rate.

### Chromatin immunoprecipitation analysis

The Chromatin Immunoprecipitation (CHIP) kit (Beyotime, Shanghai, China) was used according to the guidelines. Huh7 and MHCC97H cells were de-crosslinked using a 1% formaldehyde solution at 37 °C, followed by sonication on ice, and the lysates were immunoprecipitated overnight with BTF3 and IgG antibodies, respectively. DNA fragments were enriched by adding Protein A/G Magnetic Beads to the samples for one hour. After elution, the DNA was purified and analyzed by qRT-PCR. The PDCD2L promoter-specific primer sequences used are shown in Supplementary Table 1 (Table.S1).

### Dual-luciferase reporter assay

The PDCD2L proximal promoter sequence (corresponding to transcription start site -2000 to 0) was amplified and cloned into the pGL3-Basic plasmid. Huh7 and MHCC97H cells with control plasmid and BTF3 plasmid were inoculated into 96-well plates, and the dual-luciferase plasmids (pGL3-PDCD2L or pGL3-Basic control plasmid) were assayed using the Dual-luciferase reporter gene assay kit using lipo8000.

### Immunohistochemistry (IHC)

For IHC, paraffin-embedded, formalin-fixed HCC and adjacent non-tumor tissue sections were used after deparaffinization and hydration, and the sections were blocked for endogenous peroxidase by 3% H₂O₂ and pre-treated for 300 s by microwave heating in EDTA (ph 8.0). Afterwards, BTF3 antibody and secondary antibody were incubated (37 °C, 0. 5 h). Sections were stained by DAB and counterstained with hematoxylin. Scoring of IHC results based on H-score (Gao et al. 2020) H-SCORE = ∑ (pi × i) = (percentage of weak intensity × 1) + (percentage of moderate intensity × 2) + (percentage of strong intensity × 3).

### In vivo mouse xenograft study

Five-week-old BALB/C female nude mice were purchased from Vital River Laboratory Animal Technology. Twenty-four nude mice were randomly divided into four groups of four. The nude mice were injected subcutaneously with MHCC97H cells (5 × 10^6^) carrying the corresponding transfection plasmid. The length and width of the tumors were measured and recorded every two days. Tumor volume was calculated as V = 0.5 × (length × width^2^). The nude mice were executed 20 days after the subcutaneous injection of cells, and the subcutaneous loaded tumors were removed. The First Affiliated Hospital Ethics Committee of Zhengzhou University, China, approved the animal experiments.

### Statistical analysis

Data are expressed as mean ± SD of three independent experiments. Student’s t-tests were used for comparison between two groups, and one-way analysis of variance (ANOVA) was used for comparison between multiple groups and Bonferroni's test was used for validation. Survival analysis was calculated by the K-M method. P values < 0.05 were considered statistically significant.

## Supplementary Information

Supplementary Material 1.

Supplementary Material 2.

## Data Availability

The datasets used during the current study are available from the corresponding author on reasonable request.
