# Supplementary figures and images for: BTF3 affects hepatocellular carcinoma progression by transcriptionally upregulating PDCD2L and inactivating p53 signaling
Source: Mol Med. 2024 Dec 20;30:252. doi: 10.1186/s10020-024-01044-x (PMC11660624; doi:10.1186/s10020-024-01044-x)

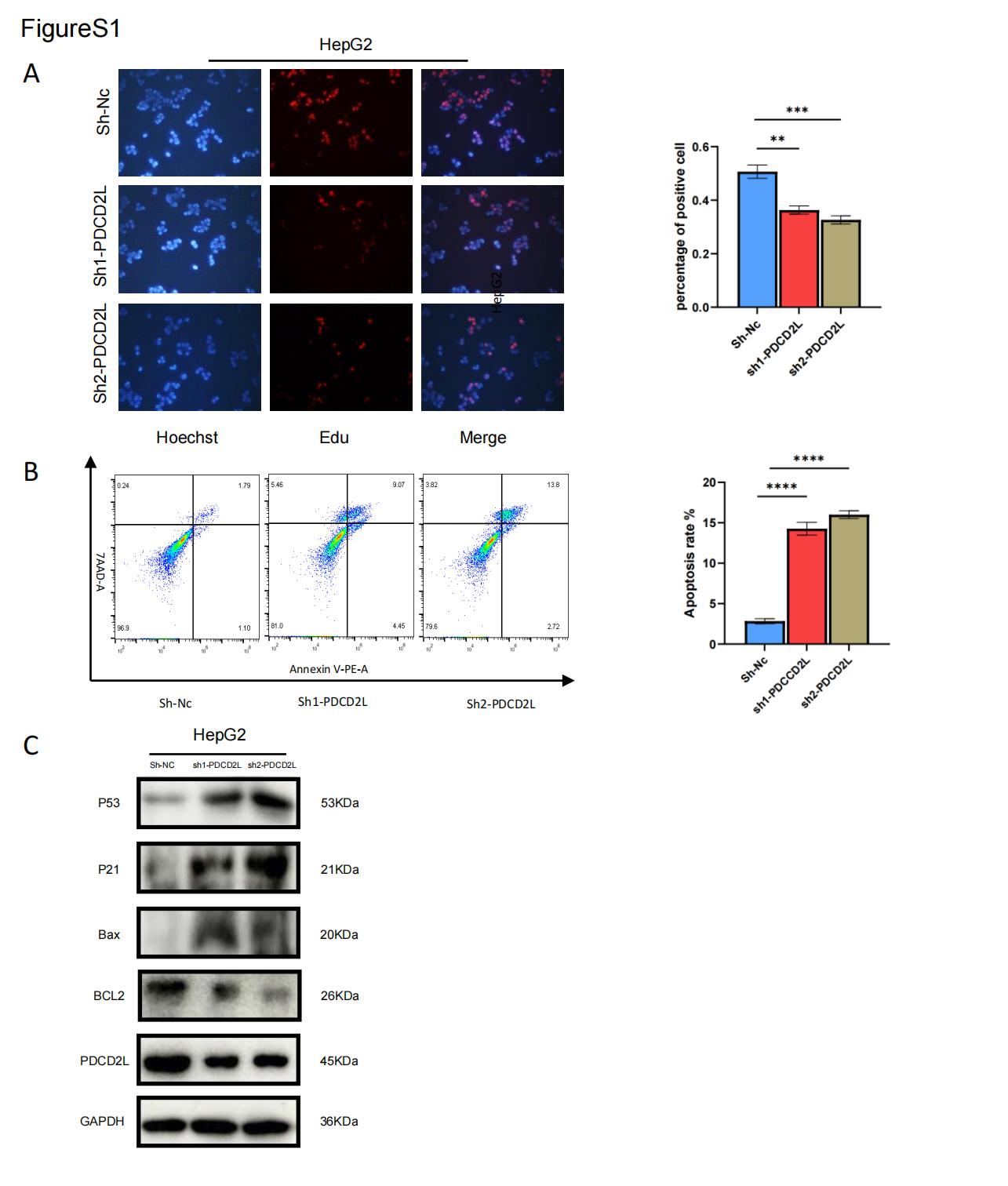

Supplement: Supplementary file 1 — Supplementary Material 1. [file 10020_2024_1044_MOESM1_ESM.jpg]
